# Experimental Study on the Mechanism of Cinnamaldehyde Ameliorate Proteinuria Induced by Adriamycin

**DOI:** 10.1155/2022/9600450

**Published:** 2022-07-23

**Authors:** Dan He, Qiang Li, Guangli Du, Shaoli Chen, Puhua Zeng

**Affiliations:** ^1^School of Basic Medical Science, Shanghai University of Traditional Chinese Medicine, Shanghai 201203, China; ^2^Hunan Academy of Traditional Chinese Medicine Affiliated Hospital, Hunan 410006, China; ^3^School of Pharmacy, Shanghai University of Traditional Chinese Medicine, Shanghai 201203, China

## Abstract

**Objective:**

Cinnamaldehyde (CA) is the main active component of Guizhi (*Cinnamomi ramulus*) to ameliorate adriamycin- (ADR-) induced proteinuria in rats. However, the underlying mechanism of CA against proteinuria remains unclear. The aim of this study was to investigate the action mechanisms of CA to treat proteinuria.

**Methods:**

13 rats were randomly selected from 78 SD rats as control group, and the other rats were injected with ADR (3 mg/kg/time) twice through tail vein on day 1 and day 8 for modeling. After modeling, the rats were randomly divided into 5 groups as follows: ADR group, ADR+CA low-dose group, ADR+CA middle-dose group, ADR+CA high-dose group and Benazepril group with 13 rats in each group. The urine of SD rats was collected for 24 h, urine protein, creatinine and urea nitrogen were detected, renal index was calculated, and HE staining and western blot were performed.

**Results:**

The 24 h urine volume and urine protein, renal function, and renal histopathology got worse significantly in the ADR group. To western blot, CA downregulated the protein expression of ACE and Ang-2 and upregulated the protein expression of ACE2 in RAS signaling pathway.

**Conclusion:**

The underlying action mechanism of CA to treat NS might mainly be achieved by regulating RAS signaling pathway.

## 1. Introduction

Nephrotic syndrome (NS) is a renal disease with clinical manifestations such as proteinuria [1]. Improper treatment can lead to the development of NS into chronic end-stage renal failure, which seriously affect people's health and life quality [2]. So far, the related etiopathogenesis of proteinuria remains unclear. Therefore, in order to provide scientific theoretical basis for the clinical treatment of proteinuria, we should actively explore the etiopathogenesis of proteinuria.

Wulingsan is a blended traditional Chinese herbal medicine specifically used for various kidney diseases and can ameliorate ADR-induced proteinuria in rats [3]. Guizhi is the key medicine of Wulingsan and plays a crucial role in the treatment of kidney diseases [4]. Cinnamaldehyde (CA), a core active ingredient of Guizhi [5], is considered as a compound with vascular endothelial protective function, etc. [6]. Based on the previous studies, we can infer that CA has an improvement effect on ADR-induced proteinuria [5]. However, its specific mechanism of action remains unclear.

To explore the potential mechanism of CA in the treatment of proteinuria, we followed the research method of He et al. [7]. And *in vivo* experiment was used to explore the action mechanisms of CA to treat proteinuria.

## 2. *In Vivo* Experiment

### 2.1. Materials

The following materials were obtained: CA: Shanghai Yuanye Bio-Technology Co., Ltd.; ADR: Shanghai Wokai Chemical Reagent Co., Ltd.; benazepril: Beijing NOVARTIS Pharmaceutical Co., Ltd.; primary antibody of ACE, ACE2, and Ang-2: Santa Cruz Biotechnology Inc.; GAPDH: Proteintech Group, Inc.; and secondary antibody: Signalway Antibody LLC.

### 2.2. Methods

13 rats were randomly selected from 78 SD rats (aged 6-8 weeks, weighing 200 ± 20 g) as control group (CON, *n* = 13), and the other 65 rats were injected with ADR twice (3 mg/kg/time) via tail vein on day 1 and 8, respectively. The total dose of ADR was 6 mg/kg, and the control group was injected with equal dose of normal saline. After modeling, 65 SD rats were randomly divided into 5 groups as follows: ADR (ADR, 6 mg/kg, *n* = 13), ADR+CA low-dose (CA-L, 26 mg/kg, *n* = 13), ADR+CA middle-dose (CA-M, 78 mg/kg, *n* = 13), ADR+CA high-dose (CA-H, 234 mg/kg, *n* = 13), and ADR+benazepril (benazepril, 12 mg/kg, *n* = 13), and 13 rats without modeling were used as the CON group. Each group was given 10 mL/kg intragastric administration of corresponding intervention drugs. The concentrations of CA in L, M, and H groups were 2.6 mg/mL, 7.8 mg/mL, and 23.4 mg/mL, respectively. The benazepril group was given 1.2 mg/mL. The CON group and ADR group were intragastric with equal volume of normal saline once a day for 28 days.

### 2.3. The Detection of 24 h Urine Volume and Urine Protein

After the last administration of the rats in all group, the urine was collected for measurement. After the collection, the urine volume of rats in each group was measured, and then, the proteinuria content was determined according to the instructions of the urine protein detection kit.

### 2.4. The Detection of Creatinine, Urea Nitrogen, and Renal Index

The rats were weighed before death, and then blood was collected through the abdominal aorta of rats. After blood collection, 5 mL of whole blood was put into the anticoagulation tube. The contents of serum creatinine and urea nitrogen were determined by the automatic biochemical analyzer. After the kidney was removed, the weight of the kidney was weighed and the renal index was obtained by comparing the kidney weight to the body weight of the rat.

### 2.5. Renal Histopathology

The left kidney was fixed in 10% formaldehyde, and the right kidney was divided into 3 parts and cryopreserved; subsequently, HE staining was performed. The pathological changes of kidney were observed through the light microscope.

### 2.6. Western Blot

Total protein from kidney tissue was extracted, and the protein concentration was measured by BCA method. After electrophoresis and membrane transfer, 15 *μ*L of sample loading was taken from each group, and separated by SDS-PAGE gel, PVDF film transfer, non-fat milk was used to block the membrane for 2 h, and then washed 3 times with TBST. The protein was incubated overnight with the following primary antibodies of ACE, ACE2, Ang-2, and GAPDH at 4°C and washed 3 times with TBST for 12 min each time. After the secondary antibody was added and incubated for 4 h, the protein was washed 3 times with TBST for 12 min each time. And the protein levels were quantified using Image Studio Ver5.2.

### 2.7. Statistical Analysis

GraphPad 8.0.1 software was used for data analysis in this study. Among them, one-way ANOVA, Kruskal-Wallis H rank-sum test, and Tukey method were used for the statistical analysis, and the difference was considered statistically significant when *p* < 0.05.

## 3. Results

### 3.1. The Analysis of 24 h Urine Volume and Urine Protein

**Figure 1:**
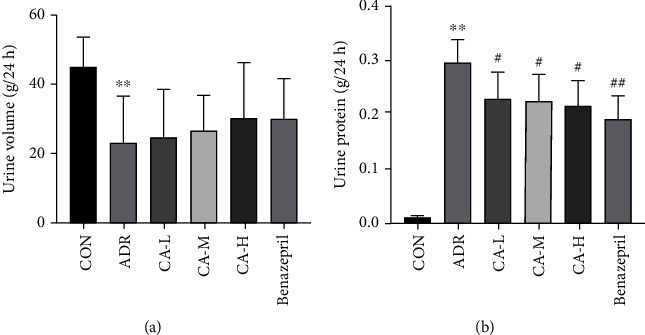
24 h urine volume (a) and urine protein (b). ^∗∗^*p* < 0.01 vs. CON group; ^#^*p* < 0.05 and ^##^*p* < 0.01 vs. ADR group.

In 24 h urine volume, the ADR group was significantly decreased than the CON group (*p* < 0.01); however, the CA-L, CA-M, CA-H, and Benazepril groups were not statistically significant compared with the ADR group (Figure 1(a)). In urine protein, the ADR group was significantly increased than the CON group (*p* < 0.01), and the CA-L, CA-M, CA-H, and benazepril groups were decreased than those in ADR group (*p* < 0.05 or *p* < 0.01) (Figure 1(b)).

### 3.2. The Analysis of Creatinine, Urea Nitrogen, and Renal Index

**Figure 2:**
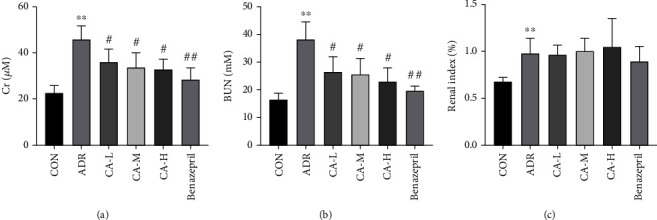
The Cr (a), BUN (b), and renal index (c). ^∗∗^*p* < 0.01 vs. CON group; ^#^*p* < 0.05 and ^##^*p* < 0.01 vs. ADR group.

In Cr, the ADR group was significantly increased than the CON group (*p* < 0.01), and the CA-L, CA-M, CA-H, and benazepril groups were decreased than ADR group (*p* < 0.05 or *p* < 0.01) (Figure 2(a)). In BUN, the ADR group was significantly increased than the CON group (*p* < 0.01), and the CA-L, CA-M, CA-H, and benazepril groups were decreased than ADR group (*p* < 0.05 or *p* < 0.01) (Figure 2(b)). In renal index, the ADR group was significantly increased than the CON group (*p* < 0.01); however, the CA-L, CA-M, CA-H, and benazepril groups were not statistically significant compared with the ADR group (Figure 2(c)).

### 3.3. The Analysis of Renal Histopathology

**Figure 3:**
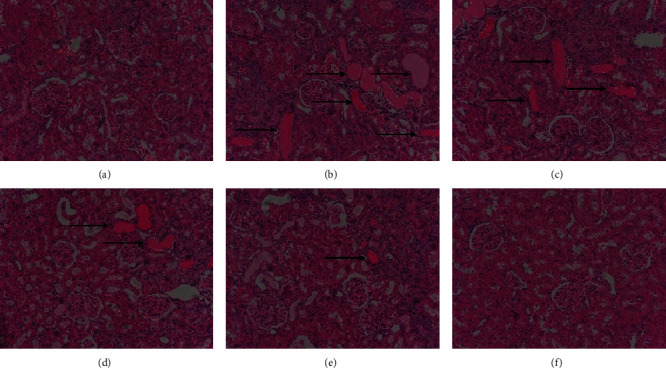
Pathological changes of kidney (HE: 200x). (a) CON group, (b) ADR group, (c) CA-L group, (d) CA-M group, (e) CA-H group, and (f) benazepril group.

The structures of glomeruli, renal capsules, and renal tubules in the CON group were intact (Figure 3(a)). In the ADR group, renal tubular epithelial cells showed degeneration and swelling, and some of them were highly dilated and varied in size and shape, with protein tubular type and inflammatory infiltration (Figure 3(b)). CA-L (Figure 3(c)) and CA-M (Figure 3(d)) group showed partial glomerular sclerosis, renal tubular dilatation, and a small amount of inflammatory infiltration, and the degree of injury was less than that of the ADR group. CA-H (Figure 3(e)) and benazepril group (Figure 3(f)) showed only a small amount of renal tubule dilatation and inflammatory infiltration.

### 3.4. The Analysis of Western Blot

**Figure 4:**
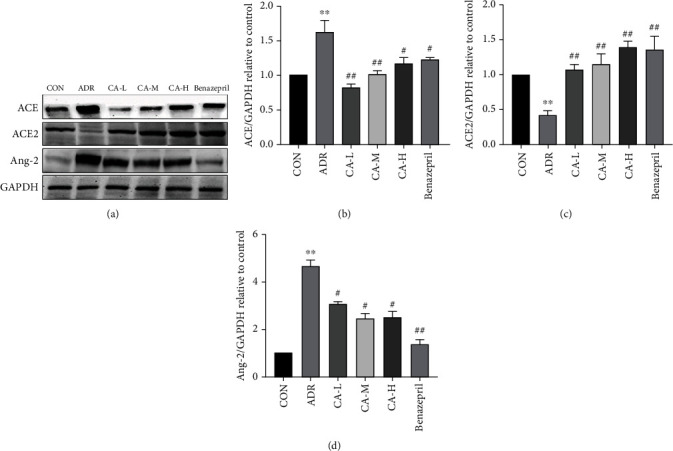
The levels of protein expression of ACE (b), ACE2 (c), and Ang-2 (d) were detected by western blotting. ^∗∗^*p* < 0.01 vs. CON group; ^#^*p* < 0.05 and ^##^*p* < 0.01 vs. ADR group.

Western blot analysis was performed to evaluate the protein expression levels of ACE, ACE2, and Ang-2 in renal tissues of rats. As shown in Figure 4(a), compared with the CON group, ACE (Figure 4(b)) and Ang-2 (Figure 4(c)) levels were significantly increased, while ACE2 (Figure 4(d)) levels were significantly decreased in the ADR group. Compared with the ADR group, ACE and Ang-2 levels were decreased in the CA-L, CA-M, CA-H, and benazepril groups, while ACE2 levels were increased.

## 4. Discussion

Renin angiotensin system (RAS) is composed of a series of peptide hormones and corresponding enzymes with the functions of regulating blood pressure and maintaining water and electrolyte balance, which is an important mechanism involved in the pathogenesis of hypertension [8]. RAS has two axes of action. One is the classical axis that composed of ACE, Ang-2 and AT1, which can activate various cellular functions and signaling pathways related to hypertension and tissue damage. The other is the reaction axis, composed of ACE2, Ang (1-7), and Mas, which acts in reverse of the classical axis [9].

The ACE and Ang-2 are the key components of the RAS classical axis. The angiotensinogen released by the liver is hydrolyzed by renin to Ang I, which is then lysed by ACE to produce Ang-2. The main effects of Ang-2 include vasoconstriction, water and sodium retention, and aldosterone release [10]. As one of the strongest vasoconstrictor substances known up to now, Ang-2 can bind to angiotensin receptors on vascular smooth muscle, thereby constricting arterioles throughout the body and raising blood pressure [11].

ACE2, a major component of the RAS reaction axis, is a homologue of ACE [12] and mainly expressed in renal vascular endothelial and tubular epithelium. ACE2 can transform Ang-2 into Ang (1-7) and antagonize a variety of pathophysiological effects that mediated by Ang-2, thus effectively playing the role of endogenous ACE inhibitor, reducing Ang-2 content, lowering blood pressure, and alleviating kidney damage [13]. Blocking RAS has a protective effect on the kidney, and the activation of classical axis can lead to progressive renal damage [14]. On the contrary, reaction axis can slow the progression of kidney disease in a variety of animal models [15].

NS is a group of clinical syndromes that characterized by massive proteinuria and edema, which results from increased permeability due to destruction of the glomerular basement membrane, and is one of the causes of end-stage renal disease [16]. The kidney plays a crucial role in the development of hypertension, which in turn can aggravate kidney damage. The interaction between the two can promote the further development of the disease [17]. For example, renal artery stenosis and renal parenchymal lesions can release large amounts of renin from juxtaglomerular cells, resulting in increased Ang-2 activity and systemic arteriole wall contraction and subsequently resulting in hypertension. In addition, renin and Ang-2 can promote the secretion of aldosterone increase, resulting in water and sodium retention, and further increase the blood volume, thereby aggravating hypertension [18]. So, hypertension is both a cause and a consequence of renal disease.

Proteinuria is the main manifestation of NS, as well as the main pathological change in rats of adriamycin-induced NS [19]. Studies show that RAS is closely related to hypertension and proteinuria [20], the occurrence of hypertension will in turn aggravate proteinuria, and lowering blood pressure can reduce proteinuria [21]. Studies have found that inhibition of RAS can reduce kidney injury and albuminuria, and Ang-2 is mainly involved in the development of the disease [22]. Jin et al. found that ACE2 knockout also led damaging effects on renal function, mainly manifested as proteinuria and inflammatory reaction [23].

CA has a variety of pharmacological activities such as regulating blood pressure [24] and alleviating vascular endothelial injury [6]. The previous study found that CA had a good protective effect on adriamycin-induced nephropathy in rats [5]. And in this study, low, medium, and high doses of CA could significantly reduce urinary protein and renal injury degree in rats of adriamycin-induced proteinuria and improve renal function simultaneously.

In order to evaluate the protein expression levels of ACE, ACE2, and Ang-2, western blot was performed. As shown in Figure 4, compared with the CON group, the levels of ACE and Ang-2 in the ADR group were significantly increased, while the level of ACE2 was significantly decreased. Compared with the ADR group, the levels of ACE and Ang-2 were decreased, while the level of ACE2 was increased in each administration group. The ADR group may have increased blood pressure due to the significantly increased ACE and Ang-2 levels, which eventually led to a large amount of proteinuria. The level of ACE2 was significantly increased in the administration group, and the blood vessels were dilated, so that proteinuria was reduced. These results confirmed our hypothesis that RAS might be the action pathway of CA in the treatment of proteinuria.

## 5. Conclusion

In summary, *in vivo* experiment was performed in this study to explore the potential mechanism of CA on proteinuria. And the results indicated that the mechanism of CA against proteinuria might be mainly through the regulation of RAS signaling pathway. Therefore, we speculate that CA may be a new and promising potential compound for the treatment of proteinuria.

## Data Availability

The data supporting the results of my study can be found in the article.
